# Energy Stored and Capacitance of a Circular Parallel Plate Nanocapacitor

**DOI:** 10.3390/nano11051255

**Published:** 2021-05-11

**Authors:** Orion Ciftja

**Affiliations:** Department of Physics, Prairie View A&M University, Prairie View, TX 77446, USA; ogciftja@pvamu.edu

**Keywords:** nanocapacitor, energy, capacitance, circular plate, dielectric thin film, 07.50.-e, 77.55.-g, 77.55.F-

## Abstract

Nanocapacitors have received a great deal of attention in recent years due to the promises of high energy storage density as device scaling continues unabated in the nanoscale era. High energy storage capacity is a key ingredient for many nanoelectronic applications in which the significant consumption of energy is required. The electric properties of a nanocapacitor can be strongly modified from the expected bulk properties due to finite-size effects which means that there is an increased need for the accurate characterization of its properties. In this work, we considered a theoretical model for a circular parallel plate nanocapacitor and calculated exactly, in closed analytic form, the electrostatic energy stored in the nanocapacitor as a function of the size of the circular plates and inter-plate separation. The exact expression for the energy is used to derive an analytic formula for the geometric capacitance of this nanocapacitor. The results obtained can be readily amended to incorporate the effects of a dielectric thin film filling the space between the circular plates of the nanocapacitor.

## 1. Introduction

Extensive research efforts in nanotechnology during the last two decades have led to great advances in the fabrication of novel materials such as carbon nanotubes, single electron transistors, nanowires, semiconductor nano dots, to mention a few, with length scales in the nanometer range [1,2,3,4,5,6,7,8,9,10,11,12,13,14,15,16,17,18]. New and improved experimental techniques have enabled the creation of various nanoparticles that can serve as fundamental building blocks in the assemblage of complex structural components in more elaborate nanoscale systems [19,20,21]. Additionally, the novel properties of artificially made nanostructures can be exploited to tailor device performance as well as enhance reliability. Nanoelectronic technologies are now fully focused on the design of nanoscale transistors that, in principle, must be integrated into nanoscale circuits (nanocircuits). A nanocapacitor is one of the fundamental elements required to design a feasible nanocircuit with the other elements being the nanoinductors and nanoresistors [22]. Therefore, the fabrication and characterization of nanocapacitors are a required step to build prototypes of functional nanocircuits.

Energy storage devices such as supercapacitors and batteries have always drawn much attention for their potential applications [23]. Conventional batteries can store substantial amounts of energy but they have the drawback of their charging time that is much longer than that of a capacitor. As a result, there is a revamped effort to fabricate capacitors with high energy storage capacity. Such capacitors are essentially parallel-plate electrostatic capacitors which can store charge on the surfaces of the two metallic conducting plates. The nanoscale counterpart of such a bulk capacitor, the nanocapacitor, has been shown to have the capability to make use of densely packed interfaces and thin films. As a result, nanocapacitors may potentially serve as the basis of next-generation electronic devices [24]. There are many methods used to fabricate nanocapacitors. However, as miniaturization reaches the nanoscale, all devices fabricated through these approaches start to show size-dependent properties and are becoming extremely difficult to characterize [25].

In this work, we introduce a model for a circular parallel plate nanocapacitor consisting of two identical circular plates placed face-to-face opposite to each other at an arbitrary separation distance. All spatial dimensions of the system are constrained to be in the nanoscale. Each of the plates of the nanocapacitor is assumed to be a perfectly two-dimensional (2D) circular disk with a radius, *R*, that measures on the nanoscale. The separation distance between the two circular plates is considered arbitrary, however, of the same order of magnitude as the radius. Given this layout, one expects the properties of this nanocapacitor to be very sensitive to the geometry and depend in a non-obvious way on the size of the circular plates and inter-plate separation distance. For such a model, we do not consider any quantum effects and/or material-dependent properties that do affect realistic experimentally manufactured nanocapacitor-related systems [26,27,28,29]. The focus of this work was to introduce a model for a nanocapacitor that would allow us to obtain an exact expression for the energy stored and/or its capacitance as a function of size and geometry. The influence of a dielectric material between the plates of the nanocapacitor can be later added to the model through the addition of a phenomenological size/thickness-dependent relative permittivity parameter. It was shown that the model that is currently investigated allows one to obtain an exact expression for the stored energy and the capacitance of such a nanocapacitor as a function of its size and geometry. It was found that the energy storage capability of the nanocapacitor when the plates are charged and its corresponding capacitance depend in a non-obvious way on the separation distance between the two circular plates.

The article is organized as follows. In Section 2, we explain the theory and the model that we use to study the nanocapacitor under consideration. In Section 3, we discuss the results obtained for the energy stored in this nanocapacitor. In Section 4, we provide an exact expression for the corresponding nanocapacitance. In Section 5, we discuss the scope of the research in more detail, elaborate on the rationale of the work and add some possible application examples. In Section 6, we briefly summarize the findings and provide some concluding remarks.

## 2. Theory and Model

**Figure 1:**
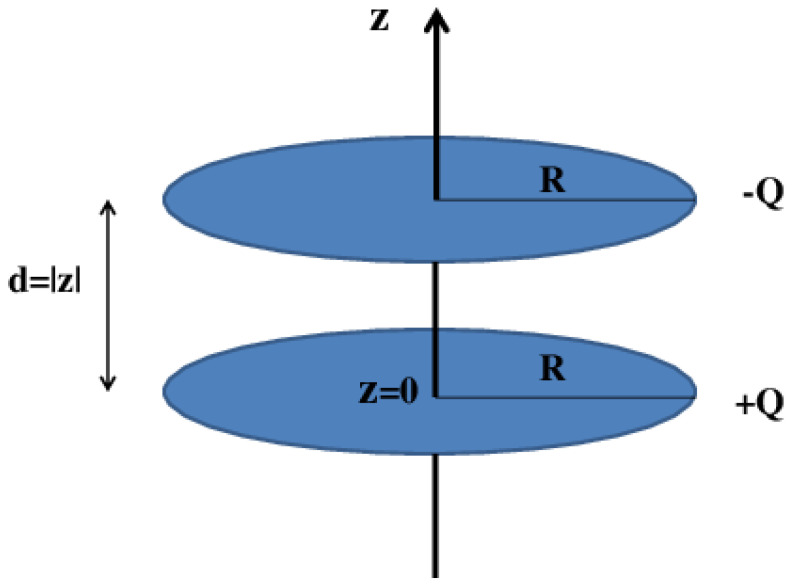
Schematic view of a circular parallel plate nanocapacitor. The two circular plates have a radius *R* and are at a distance, d=|z|≥0 apart. The respective ±Q charge of each of the circular plates is assumed to be uniformly spread over the corresponding surfaces.

The schematic model for a circular parallel plate nanocapacitor is depicted in Figure 1. The circular plates have an identical radius *R* and contain, respectively, a charge of +Q and −Q. It is assumed that charge is uniformly distributed over each of the two circular plates. The respective uniform surface charge densities are denoted as ±σ where $\sigma =Q/(\pi \;R^{2})$ represents the surface charge density of the positively charged circular plate. The system of coordinates is so chosen that the two circular plates lie parallel, opposite to each other, and perpendicular to the *z* direction. The positively charged circular plate, denoted 1, lies in the z=0 plane. The negatively charged circular plate, denoted 2, lies at some arbitrary *z* plane. The origin of the system of coordinates coincides with the center of circular plate 1. The separation distance between the two parallel circular plates is denoted as d=|z|≥0.

For this choice of the coordinative system, the elementary charges in the circular plate 1 are all confined to the domain, $D_{1}$ written as

(1) $$D_{1}:\left\{\begin{array}{c} 0\leq \sqrt{x_{1}^{2}+y_{1}^{2}}\leq R\\ z_{1}=0\;. \end{array}\right.$$

The elementary charges in the circular plate 2 are all confined in the domain, $D_{2}$ given as

(2) $$D_{2}:\left\{\begin{array}{c} 0\leq \sqrt{x_{2}^{2}+y_{2}^{2}}\leq R\\ z_{2}=z\;. \end{array}\right.$$

It is assumed that any two arbitrary elementary charges, $dq_{1}$ and $dq_{2}$, interact with each other via the standard Coulomb interaction potential, $k_{e}\;dq_{1}\;dq_{2}/\left|{\vec{r}}_{1}-{\vec{r}}_{2}\right|$ where $k_{e}$ is Coulomb’s electric constant and $|{\vec{r}}_{1}-{\vec{r}}_{2}|$ is the separation distance between the pair of elementary charges.

The energy stored in the nanocapacitor will depend in a non-trivial way on the geometry of the model since there are no assumptions made about *R* and *d*. In turn, this should lead to a finite-size geometric capacitance that should resemble the macroscopic one only for d≪R, or otherwise, will be strikingly different. At this point, we take the opportunity to clarify that we use the term “macroscopic capacitor” as opposed to “nanocapacitor” to describe any device where the standard formula of a parallel plate capacitor, $C_{m}=\epsilon_{0}\;A/d$, is used to describe its capacitance (in free space) where *A* is the area of the plates and *d* is their separation distance. In reality, for any capacitor, micro or macro, the simple textbook formula is only a valid approximation when the separation distance of the plates is much smaller than their radius (for a circular plate) or length (for a square plate). To start with, let us write the total electrostatic energy stored in the nanocapacitor as

(3) $$U=U_{11}+U_{22}+U_{12}\;,$$

where $U_{11}$ ($U_{22}$) represent, respectively, the Coulomb self-energy stored in circular plate 1 (2) while $U_{12}$ represents the Coulomb electrostatic interaction energy between the two circular plates of the nanocapacitor separated by an arbitrary distance. The positive Coulomb self-energy of each of the two circular plates is identical. Thus, one can write the total electrostatic energy of the circular parallel plate capacitor as

(4) $$U=2\;\;U_{11}+U_{12}\;.$$

The Coulomb self-energy of a uniformly charged circular plate, namely, a uniformly charged disk is readily available, in the literature [30,31,32] and one way to write it is:

(5) $$U_{11}=\frac{8}{3\;\pi }\;\frac{k_{e}\;Q^{2}}{R}.$$

The calculation of the negative interaction energy, $U_{12}$, is more difficult from a mathematical point of view. For this reason, we explain all the necessary steps in some detail in order to better understand the derivation of the final result. On starts with the following integral expression:

(6) $$U_{12}=-k_{e}\;\sigma^{2}\int_{D_{1}}d^{2}\rho_{1}\int_{D_{2}}d^{2}\rho_{2}\;\;\frac{1}{|{\vec{r}}_{1}-{\vec{r}}_{2}|}\;,$$

where ${\vec{\rho }}_{i}=\left(x_{i},y_{i}\right)$(i=1,2) are 2D position vectors while ${\vec{r}}_{1}=\left(x_{1},y_{1},z_{1}=0\right)$ and ${\vec{r}}_{2}=\left(x_{2},y_{2},z_{2}=z\right)$. To facilitate the calculation of the energy expression in Equation (6), one writes that quantity as

(7) $$U_{12}=-\sigma \int_{D_{2}}d^{2}\rho_{2}\;V\left({\vec{r}}_{2}\right)\;,$$

where:

(8) $$V\left({\vec{r}}_{2}\right)=k_{e}\;\sigma \int_{D_{1}}d^{2}\rho_{1}\;\frac{1}{|{\vec{r}}_{1}-{\vec{r}}_{2}|}\;,$$

represents the electrostatic potential due to the circular plate 1 at the location of circular plate 2. Note that: $|{\vec{r}}_{1}-{\vec{r}}_{2}{|}^{2}=|{\vec{\rho }}_{1}-{\vec{\rho }}_{2}{|}^{2}+{\left|z\right|}^{2}$. The form of Equation (7) is convenient since a general formula for the electrostatic potential created by a uniformly charged disk at some arbitrary point in space is available in the literature [33]. In fact, one may start from that point and calculate quite generally the electrostatic interaction energy between any two coaxial parallel uniformly charged disks with different radii and different charges [34]. A general expression that applies to such a situation for two uniformly charged disks with respective charges *Q* and $Q^{\prime }$ (and radii, *R* and $R^{\prime }$) is given in Ref. [34]. For the current case of two circular parallel plates in a nanocapacitor, one has $Q^{\prime }=-Q$ and $R^{\prime }=R$. By combining Equations (17) and (19) in Ref. [34] one has:

(9) $$U_{12}\left(a\right)=-\left\{-2\;a+\frac{2\;a}{3\;\pi }\left[\left(4-a^{2}\right)\;E\left(-\frac{4}{a^{2}}\right)+\left(4+a^{2}\right)\;K\left(-\frac{4}{a^{2}}\right)\right]\right\}\;\frac{k_{e}\;Q^{2}}{R}\;,$$

where the parameter a=|z|/R≥0 is explicitly shown as an argument of the interaction energy and:

(10) $$K\left(m\right)=\int_{0}^{\pi /2}\frac{d\;\theta }{\sqrt{1-m\;\sin^{2}\left(\theta \right)}}\;,$$

and:

(11) $$E\left(m\right)=\int_{0}^{\pi /2}d\;\theta \;\sqrt{1-m\;\sin^{2}\left(\theta \right)}\;,$$

are, respectively, the complete elliptic integrals of the first and second kind. The notation adopted above for the complete elliptic integrals of the first and second kind follow that of Ref. [35].

## 3. Energy

To calculate the total electrostatic energy of the circular parallel plate nanocapacitor, we substitute the results from Equations (5) and (9) into the expression provided by Equation (4). This leads to an exact analytical expression for the total energy stored in a circular parallel plate nanocapacitor with free space between the plates written in compact form as

(12) $$U\left(a\right)=F\left(a\right)\;\frac{k_{e}\;Q^{2}}{R}\;,$$

where F(a) is the following auxiliary function:

(13) $$F\left(a\right)=\left\{\frac{16}{3\;\pi }+2\;a-\frac{2\;a}{3\;\pi }\left[\left(4-a^{2}\right)\;E\left(-\frac{4}{a^{2}}\right)+\left(4+a^{2}\right)\;K\left(-\frac{4}{a^{2}}\right)\right]\right\}\;\frac{k_{e}\;Q^{2}}{R}\;.$$

Note that, from this point on, the parameter a=|z|/R≥0 is explicitly shown in the argument of the total energy expression displayed in Equation (12). The reader can verify that:

(14) U(a=0)=0;F(a=0)=0.

Therefore, one can expand the expression for F(a) in Equation (13) in a power series of about a=0 to obtain the dependence of U(a) for a small *a*. The result to the lowest order (linear order) of a parameter, *a*, is:

(15) $$U_{linear}\left(a\right)=2\;a\;\frac{k_{e}\;Q^{2}}{R}\;,$$

where a=|z|/R and |z| is the separation distance between the two circular plates of the capacitor. One can check that $U_{linear}\left(a\right)$ in Equation (15) is identical to the expression for the energy stored in a macroscopic ideal parallel plate capacitor constructed from two very large uniformly charged circular plates of area *A* in free space:

(16) $$U_{m}=\frac{Q^{2}}{2\;C_{m}}\;,$$

where the capacitance (in free space) is:

(17) $$C_{m}=\epsilon_{0}\;\frac{A}{d}=\epsilon_{0}\;\frac{\pi \;R^{2}}{\left|z\right|}\;.$$

The equivalence of $U_{linear}\left(a\right)$ in Equation (15) to $U_{m}$ in Equation (16) is easy to verify if one starts from Equation (16) and rewrites it in terms of $k_{e}Q^{2}/R$ and a=|z|/R without forgetting that the Coulomb’s electric constant is $k_{e}=1/\left(4\;\pi \;\epsilon_{0}\right)$.

**Figure 2:**
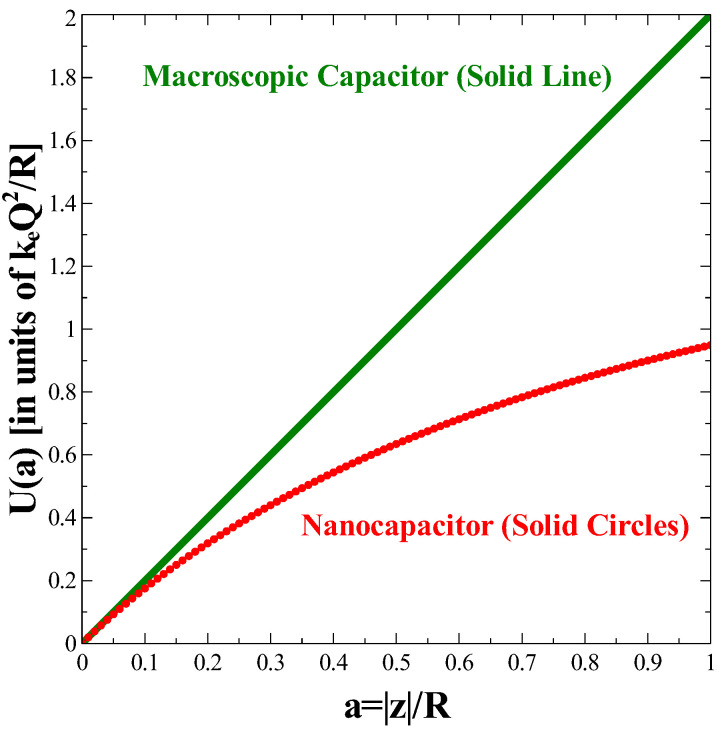
Energy stored in a circular parallel plate nanocapacitor, U(a), in units of $k_{e}\;Q^{2}/R$ as a function of the parameter a=|z|/R (solid circles) where |z| is the separation distance between the two identical circular parallel plates placed opposite to each other and *R* is their radius. The circular plates contain, respectively, a charge of ±Q. Charge is uniformly spread over each of the two circular plates resulting in uniform surface charge densities, ±σ. The exact result, U(a), is compared to the approximate expression, $U_{linear}\left(a\right)$ which represents the energy of an ideal macroscopic circular parallel plate capacitor (solid line).

Figure 2 shows the dependence of the energy, U(a), stored in a circular parallel plate nanocapacitor as a function of parameter a=|z|/R (solid circles) in conjunction with $U_{linear}\left(a\right)$ (solid line), its counterpart for a macroscopic capacitor. The energies are expressed in units of $k_{e}\;Q^{2}/R$. It can be seen that the amount of energy stored in the finite-size nanocapacitor, U(a), is substantially different from that obtained when the formula of a macroscopic capacitor $U_{linear}\left(a\right)$ is used. The differences are sizable even when the separation distance between the plates is of the order of |z|/R∝10%.

In fact, we considered this scenario in more detail, and in order to verify how reasonable the approximation is, we calculated the relative difference between the linearized approximate values (for a macroscopic bulk capacitor) and the exact values of the energy of the nanocapacitor:

(18) $$\left|\frac{U_{linear}\left(a\right)-U\left(a\right)}{U(a)}\right|\;.$$

We checked that the relative energy difference was slightly more than 14% for a value of a=|z|/R=0.1. This means that the linearized expression of the energy, namely the expression for the energy of a macroscopic capacitor, is only reliable for very small separation distances between the plates (note that, for a=0.05, the relative energy difference is quite sizable at about 8%).

Obviously, in free space, the energy stored in the nanocapacitor is electrostatic in origin and depends only on the geometry of the system. Within the framework of this model, one would account for the presence of a dielectric material between the plates by introducing a phenomenological size/thickness-dependent relative nanopermittivity parameter in the expression for the stored energy. As shown in recent work [26], the nanopermittivity parameter of very thin dielectric films is different from the bulk value and should be determined experimentally as a function of size and/or the thickness of the dielectric film in a case-by-case basis.

## 4. Capacitance

The expression for the size-dependent geometric capacitance of the circular parallel plate nanocapacitor is extracted from the formula of the energy written as a function of the charge and capacitance:

(19) $$C\left(a\right)=\frac{Q^{2}}{2\;U(a)}\;,$$

where U(a) is given from Equations (12) and (13). One uses Equations (12) and (13) to write C(a) as:

(20) $$C\left(a\right)=\epsilon_{0}\;R\;\frac{2\;\pi }{F(a)}\;,$$

where $\epsilon_{0}=1/\left(4\;\pi \;k_{e}\right)$ and F(a) is given from Equation (13). The expression in Equation (20) applies to a circular parallel plate nanocapacitor in free space. One can extend the result in Equation (20) to the case when there is a dielectric material between the plates by amending the formula in Equation (20) the following way:

(21) $$C_{\epsilon }\left(a\right)=\epsilon \left(a\right)\;\epsilon_{0}\;R\;\frac{2\;\pi }{F(a)}\;,$$

where ϵ(a) represents the relative nanopermittivity of a given dielectric film. The value of this parameter is expected to be size/thickness-dependent and different from the bulk, given that properties of a typical material in the nanoscale do not mirror the properties of the same material in the bulk regime. For example, it has been seen experimentally that the relative permittivity of a h-BN thin film is almost twice higher than that of its bulk BN counterpart [26]. In the present model, the role played by a dielectric film in the nanocapacitor is included in a conventional way by adding a phenomenological size/thickness-dependent relative nanopermittivity parameter in the expression of the capacitance. This means that this model may be considered as a genuine candidate to explain experiments where one notices an increase in the capacitance as a result of the decrease in the thickness of the nanocapacitor.

Note that the statement above is self-evident for all cases when the capacitance of a nanocapacitor is dominated by geometric effects. However, it is not the norm when it comes to many nanocapacitors whose properties are dominated by quantum effects. In these cases, the expected behavior is a decrease in the capacitance as a result of the decrease in the thickness of the dielectric film inside the plates. Recent experiments for a nanocapacitor made of graphene square plates/electrodes and h-BN dielectric films showed an increase in capacitance with decrease in thickness [26]. For this reason, this so-called anomalous size-dependent increase in capacitance was quickly attributed to subtle quantum effects exhibited by these materials. Note that this type of behavior is the same as that exhibited by the geometric capacitance of the model in Equation (21) as well as any other variant of such model including the case of a nanocapacitor with two square plates instead of circular plates.

## 5. Discussions

At this juncture, it is important to remark that the calculation of the electrostatic energy stored and/or capacitance of a parallel-plate capacitor is a long-standing problem in potential theory that has been addressed by many authors [36]. To the best of our knowledge, an exact analytic solution to the problem (the one that makes the system an equipotential) does not seem possible. There have been reports from few authors that have obtained such a solution, but sooner or later, all these attempts have been found faulty. For example, a recently proposed analytic solution by Atkinson et al. [37] was shown to be incorrect by Hughes [38]. With few words, an exact solution to this celebrated problem in potential theory (identifying the solution that makes the system an equipotential) does not seem possible, in the sense that to this date, no explicit analytical solution that have been reported are acceptable. To the best of our knowledge, the closest that one comes to an analytic solution is by using Love’s integral expressions [39] for the potential and related quantities, which are then solved numerically. For example, numerical values for the potential in the vicinity of a parallel plate capacitor were calculated using both the Love integral-equation method and a relaxation method [40]. Similarly, the capacitance of the circular parallel plate capacitor has been numerically calculated by several authors using various computational techniques [41,42,43,44,45].

The advantage of the model reported in this work is that it allows us to obtain an exact analytic expression for the electrostatic energy stored and/or capacitance. We are certainly not claiming that energy and/or capacitance have never been computed before. On the contrary, as we already explained, there are several works in this direction, but all of them are numerical calculations [36]. Another advantage of the model that we report is its simplicity. Potential theory problems of this nature are very difficult to solve. Calculating the equilibrium charge distribution on the two plates of the capacitor, namely obtaining the precise analytic form of the surface charge density that leads to an equipotential surface (same potential all over the surface) is an unsolvable analytical problem. A single circular disk/plate represents a rare example for which this problem is analytically solvable. The equilibrium surface charge density of a charged conducting disk is different from that of a uniformly charged disk, but the energy stored in such a circular disk does not differ much from its uniformly charged counterpart. In fact, we found that the relative energy difference between them is about 8% [32]. Based on these general physical considerations, we expected that the results for the electrostatic energy stored and/or capacitance obtained from our model would likely be not very different from this order of accuracy if compared to numerical values found for the same system.

As is known, capacitors play an important role in integrated circuits such as storing electrical energy and blocking direct current while allowing alternating currents to propagate. Nanocapacitors, for instance those based on graphene layers as plates, are becoming critical for advanced electric energy storage and for building nanoelectronic circuits. The model in this work can be directly applied to predict the capacitance of nanocapacitors made of graphene plates and hexagonal boron nitride (h-BN) films (as the dielectric material filling the space between the two plates). Such a system can achieve superior capacitor properties. Furthermore, the thickness can be experimentally tuned up to the thinnest possible value, essentially consisting of only monolayer materials: h-BN dielectric film with graphene electrodes [26]. In fact, we argued in a recent work [46] that the anomalous size-dependent increase in capacitance with a decrease in thickness seen in this family of h-BN/graphene nanocapacitors [26] is consistent with conventional electrostatic principles and can be explained without appealing to quantum effects. The effects of the differences on the geometry of plates, namely a circular plate versus a square plate, are expected to be small as long as the plates have the same area.

Our results for the stored electrostatic energy and/or capacitance of a circular parallel plate nanocapacitor may provide a sound theoretical basis to understand various microelectromechanical systems (MEMS). Applications of MEMS technology [47,48] is centered around sensors and actuators. Generally speaking, sensors provide a way to monitor some physical variables. On the other hand, actuators take an input control signal (such as voltage or current) and produce a force or torque to generate motion. As a basic example, reconsider the circular parallel plate nanocapacitor model of this work. If the distance between the two plates changes as a result of an external force, the overall capacitance also changes. The change of capacitance can be exactly calculated in this model. The value is expected to be very accurate since it represents a difference of two quantities calculated under same conditions. With other words, although the assumption of uniform charge distribution in each of the plates may introduce some error, the difference of two capacitances for two different separation distances will tend to be very accurate since these errors are systemic and will tend to cancel out. As a result, by calculating $C_{\epsilon }\left(a_{2}\right)-C_{\epsilon }\left(a_{1}\right)$, one obtains a very accurate sense of the force. In fact, this principle forms the basis for the electrostatic sensing of position when a parallel plate capacitor is used as an actuator. Assume that the bottom plate is held fixed, while the top plate is suspended by an ideal elastic spring that is free to move. One may calibrate the system so that the spring is initially underformed. This means that a variation of capacitance (in a charge-controlled actuator) provides us information on the elastic force exerted from the spring on the top plate, which in turn, provides a sensing of position. The expected high accuracy of the model used in this application can be very useful to characterize various electrostatic sensors and actuators that are found in a wide array of MEMS-based devices.

## 6. Conclusions

We introduced a model for a circular parallel plate nanocapacitor consisting of two identical uniformly charged circular plates opposite to each other at an arbitrary separation distance. It is assumed that both the radii of the circular plates and their separation distance are finite and of the same order of magnitude. An analytic result for the total energy stored due to such a nanocapacitor has been found. The final result is expressed in terms of an auxiliary function that depends on a single dimensionless parameter and can be readily evaluated. As an example for the utility of our result, the energy stored in the nanocapacitor was calculated not only generally, but also when the two electrodes are in very close proximity to each other. The result in the latter case was found to converge to the familiar expression for the energy stored in a macroscopic capacitor. As a second example, we graphically displayed the relative energy difference between the exact expression and its macroscopic counterpart showing that the macroscopic formula should be used with extreme caution and only when the spacing between the two circular plates is very small, in the order of less than 5% relative to the radius. The exact analytic formula shown in Equation (13) can help understand how the energy is stored in multi-layered capacitive nanostructures with circular symmetry [49,50,51,52,53].

The expression for the energy stored in a circular parallel plate nanocapacitor was used to derive an analytic formula for the corresponding nanocapacitance of the system. The initial result for the nanocapacitance derived under the assumption of free space between the two circular plates can be extended to a more general case scenario where such a space between the plates is filled with a dielectric film. For the given lengths in the nanoscale, it is expected that the electric properties of the very thin dielectric film will differ from its corresponding bulk counterpart as already seen in experiments with graphene boron nitrade nanocapacitors [26]. For this reason, one must assume a phenomenological size/thickness-dependent relative nanopermittivity for the dielectric film and extract its value experimentally.

At this juncture, we also point out that the results obtained thus far can also be helpful to gauge the accuracy of various theoretical approximations and numerical methods used to study the properties of systems with circular symmetry. This situation may apply to certain 2D compositions formed in semiconductor quantum dots, heterostructures or thin films [54,55,56,57] which lead to the creation of a two-dimensional electron gas (2DEG) system. In particular, the physics of quantum Hall effect (QHE) has at its heart the model of a 2D system of electrons confined in a neutralizing background represented by a uniformly charged circular disk lying on the same plane as the layer of electrons [58,59,60,61,62,63]. A tweak of this model in the context of QHE studies would be to consider the 2D layer of electrons as being space separated at some distance above the depleted positively charged jellium disk region (“the donor charge” region). In this setup, the Hartree term in a Hartree–Fock method [54] would result in a capacitive-like effect similar to the one presently studied.

## Data Availability

The data presented in this study are available upon request from the author.

## Abbreviations

The following abbreviations are used in this manuscript:

2D	Two-dimensional
MEMS	Micro-electromechanical systems
2DEG	Two-dimensional electron gas
QHE	Quantum Hall effect
